# Xue-Fu-Zhu-Yu capsule in the treatment of qi stagnation and blood stasis syndrome: a study protocol for a randomised controlled pilot and feasibility trial

**DOI:** 10.1186/s13063-018-2908-9

**Published:** 2018-09-21

**Authors:** Haoqiang He, Guang Chen, Jialiang Gao, Yu Liu, Chenhao Zhang, Chao Liu, Hongzheng Li, Qingyong He, Jun Li, Jie Wang

**Affiliations:** 1 Department of Cardiology, Guang’anmen Hospital, China Academy of Chinese Medical Sciences, Beijing, 100053 China; 2 Beijing University of Chinese Medicine, Beijing, 100029 China; 3 Department of Acupuncture, Huguosi TCM Hospital Affiliated to Beijing University of Chinese Medicine, Beijing, 100035 China; 4 EmergencyDepartment, Wangjing Hospital, China Academy of Chinese Medical Sciences, Beijing, 100102 China

**Keywords:** Xue-Fu-Zhu-Yu capsule, Qi stagnation and blood stasis syndrome, Chinese medicine, Randomised clinical trial

## Abstract

**Background:**

Qi stagnation and blood stasis syndrome (QS&BSS) is one of the common *Zhengs* in traditional Chinese medicine (TCM), which manifests as various symptoms and signs, such as distending pain or a tingling sensation in a fixed position. In recent years, a number of clinical trials have focused on the effectiveness and safety of XFZYC in patients with a QS&BSS subtype disease, such as coronary heart disease, hyperlipidaemia, ischaemic cerebrovascular disease, gastritis, dysmenorrhoea, or arthritis, in terms of the outcomes of relevant diseases. However, there is lack of evidence of the effects of XFZYC in patients with QS&BSS with different diseases, focusing on the outcomes of *Zhengs*.

**Methods/Design:**

A randomised, controlled, pilot and feasibility trial will be employed in this study, using a 7-week study period. Participants will be recruited from Guang’anmen Hospital, Huguosi TCM Hospital, Wangjing Hospital in China. One hundred and twenty participants will be randomised to a treatment group (Xue-Fu-Zhu-Yu Capsule (XFZYC)) and placebo group in a 1:1 ratio. Participants included in the study must be diagnosed with Qi stagnation and blood stasis syndrome criteria. The outcome measurements will include the traditional Chinese medicine patient-reported outcome (PRO) scale for QS&BSS, the single symptom and sign scale of QS&BSS, and the pain scale of QS&BSS. The clinical data management system (http://www.tcmcec.net/) will be used to collect and manage the data. Quality control will be used, according to Good Clinical Practice (GCP).

**Discussion:**

Previous studies were expected to evaluate whether the addition of XFZYC to standard routine treatment would enhance the treatment effectiveness and improve the biomedical parameters pertaining to relevant disease. However, this trial is focused on the outcome of *Zhengs*, and we chose a range of outcome measurements to assess the improvement of relevant symptoms and signs. This trial is the first study designed to define and optimise the outcome measurements of *Zhengs* of XFZYC in the treatment of patients with QS&BSS.

**Trial registration:**

ClinicalTrials.gov, NCT03091634. Registered on 12 August 2018. Release date 6 May 2017.

**Electronic supplementary material:**

The online version of this article (10.1186/s13063-018-2908-9) contains supplementary material, which is available to authorized users.

## Background

Traditional Chinese medicine (TCM) has experienced a long history and shaped its own theory. Within the framework of TCM theory, TCM practitioners always prescribe herbal medicines and acupuncture formulae based on specific diseases and *Zhengs* [1]. What are *Zhengs*? They can be not only a subtype disease but also a type of common symptom occurrence of different diseases. Specifically, TCM practitioners always identify *Zhengs* by recognising a slight difference in the same symptoms of the same disease. For example, chest pain is a common symptom of coronary heart disease, but this symptom could be divided into several subtypes based on the description of the pain experienced. Distending pain and stabbing pain are regarded as one subtype and type of *Zhengs*, which is named Qi stagnation and blood stasis syndrome (QS&BSS, also known as Qi-Zhi-Xue-Yu Zheng) and is treated differently from other *Zhengs*. In this way, the differentiation of *Zhengs* makes TCM treatment become individualised in some sense. On the other hand, when specific similar symptoms arise in various diseases, patients with different diseases could be treated by the same TCM therapy according to the theory of TCM, which has been accepted by the Consolidated Standards of Reporting Trials (CONSORT) for Chinese Herbal Medicine Formulas 2017 [2]. For instance, distending pain and stabbing pain in some cases might be one of the main symptoms of stable angina, stomach ache, dysmenorrhoea and other conditions. Therefore, this common symptom in different diseases could receive a complementary treatment using the same TCM therapy. Although this treatment approach allows TCM formulae to have a wide range of application and has been employed by TCM doctors for thousands of years, more specific evidence should be accumulated to support this type of approach and its underlying theory.

From the perspective of TCM, Qi and blood are considered to be the fundamental substances constituting the human body, and the material basis for human health and diseases. Therefore, QS&BSS is one of the common *Zhengs* in TCM and manifests as various symptoms and signs, such as distending pain or a tingling sensation in a fixed position, irritability or depression, dim complexion, lumps on the body, blood spots under the skin, unsmooth or string-like pulse, and purplish tongue or petechiae on the tongue. In fact, Qi stagnation and blood stasis are common pathological conditions that occur in the human body several times in a human’s life. Moreover, QS&BSS is associated with over 50 diseases, including coronary heart disease, hypertension, cerebral infarction, gastritis, arthritis, dysmenorrhoea, chronic pelvic infection, skin disease, and cancer [3–11].

There are several types of TCM therapies that are used for treating QS&BSS, including Chinese herbal medicine (CHM), acupuncture, moxibustion, cupping, massage and Qigong therapy. Xue-Fu-Zhu-Yu Capsule (XFZYC), one of the Chinese herbal medicine (CHM) drugs, could perform the function of promoting Qi and activating blood to relieve the symptoms of QS&BSS; its use was approved by the China Food and Drug Administration (CFDA) in 2002. The components of the capsule include the following: *Radix Bupleuri* (chaihu), *Angelica Sinensis* (danggui), *Radix Rehmanniae Recens* (shengdihuang), *Radix Paeoniae Rubrathe* (chishao), *Carthamus Tinctorious* (honghua), peach kernel (taoren), *Fructus Aurantii* (zhiqiao), *Radix Liquiritiae* (gancao), *Ligusticum Wallichii* (chuanxiong), *Radix Achyranthis Bidentatae* (niuxi), and *Platycodon grandiflorum* (jiegeng). XFZYC has been used for the treatment of QS&BSS since it was recorded in the book *Correction on Errors in Medical Classics* 200 years ago. In recent years, a number of clinical trials have focused on the effectiveness and safety of XFZYC in patients with QS&BSS subtype diseases, such as coronary heart disease, hyperlipidaemia, ischaemic cerebrovascular disease, gastritis, dysmenorrhoea, and arthritis [5, 7, 8, 12–14], in terms of the outcomes of relevant diseases. However, evidence for the use of XFZYC to address the *Zhengs* in patients with QS&BSS is lacking. To define and optimise an outcome design for a future, adequately powered, definitive trial to evaluate the effectiveness and safety of XFZYC in the treatment of QS&BSS, a multicentre, randomised pilot trial will be undertaken.

## Methods/Design

### Trial objective

The primary objective of this trial is to assess the acceptability and feasibility of the outcome measures as methods to evaluate the effectiveness of the CHM in terms of *Zhengs* within definitive trials.

### Research design

This study is a multicentre, prospective, double-blind, randomised, controlled pilot trial. This study design follows the international recommendations for interventional trials (see the standard protocol items: recommendation for interventional trials (SPIRIT) checklist in Additional file 1). Additional file 2 provides a brief overview of our design. This pilot and feasibility trial will be reported using the CONSORT 2010 statement extension for randomised pilot and feasibility trials as a reference [15].

### Registration

The trial was registered at Clinical Trials.gov (ID, NCT03091634). The study will be conducted in compliance with the Declaration of Helsinki and Good Clinical Practice (GCP) guidelines.

### Settings and participants

We will recruit participants in two ways. First, the study information will be advertised on hospital notice boards and the WeChat application programme, and interested participants can register for this trial by telephone with the researcher or through WeChat. Second, the clinical staff at outpatient clinics will scan and identify potential participants who will then be approached with the study information. These two methods will be followed up by a screening visit with the researchers. A total of 120 patients will be enrolled at one of the following three hospitals in Beijing, China: (1) Guang’anmen Hospital, China Academy of Chinese Medical Sciences, (2) Huguosi TCM Hospital, Beijing University of Chinese Medicine, and (3) Wangjing Hospital, China Academy of Chinese Medical Sciences.

### Diagnostic criteria

#### Traditional Chinese medicine diagnostic criteria for QS&BSS

To avoid the subjectivity associated with expert syndrome differentiation, a traditional diagnostic method, both TCM syndrome differentiation diagnostic criteria for QS&BSS and the TCM syndrome diagnostic scale for QS&BSS will be used in the trial. A diagnosis of QS&BSS should satisfy the following three TCM diagnostic criteria simultaneously:

- Expert syndrome differentiation: two deputy chief physicians will make an independent diagnosis by syndrome differentiation. If the patient is diagnosed as having QS&BSS by both physicians, he/she could be enrolled. If the diagnosis is different between the two physicians, another deputy chief physician will contribute to the final diagnosis.
- TCM syndrome differentiation diagnostic criteria for QS&BSS: primary symptoms and signs are (1) distending pain; (2) tingling sensation; (3) pain in a fixed place; (4) pain aggravated by touch; (5) lumps in the body; (6) irritability; and (7) depression.
  Secondary symptoms and signs are (1) loss of appetite; (2) dim complexion; and (3) blood spots under the skin. Tongue manifestation involves (1) purplish tongue and (2) petechiae on the tongue.
  Pulse condition involves (1) unsmooth pulse and (2) string-like pulse.
  A diagnosis of QS&BSS should include more than two primary symptoms and signs; more than one secondary symptom and sign; and one tongue presentation or pulse presentation.
- TCM syndrome diagnostic scale for QS&BSS: the TCM syndrome diagnostic scale for QS&BSS will be used to assist with the diagnosis. When the total score is greater than or equal to 20, the patient could be diagnosed with QS&BSS (See Additional file 3: Table S1).

#### Disease diagnostic criteria

The diagnostic criteria for coronary heart disease are based on the guidelines for the diagnosis and management of patients with stable ischaemic heart disease (2012) [16]; chronic gastritis is based on the consensus of chronic gastritis in China (Shanghai, 2012) [17]; cerebral infarction is based on the guidelines for the early management of patients with acute ischaemic stroke (2018) [18]; dysmenorrhoea is based on primary dysmenorrhoea consensus guidelines (2017) [19]; and arthritis is based on the Chinese guidelines for the diagnosis and management of osteoarthritis (2018) [20].

##### Inclusion criteria

The inclusion criteria are the following:

- Meets the QS&BSS criteria
- One of the five aforementioned diagnostic diseases
- Patient is 18 to 65 years of age
- Voluntarily provided written informed consent

##### Exclusion criteria

The exclusion criteria are the following:

- Diagnosis of acute myocardial infarction, acute phase of cerebral infarction, aortic dissection or other critical illness
- Poorly controlled hypertension (systolic pressure > 160 mm of mercury (mmHg) or diastolic blood pressure > 100 mmHg), severe heart failure or severe arrhythmia (atrial fibrillation, atrial flutter, ventricular tachycardia, paroxysmal type II atrioventricular block and complete bundle branch block)
- Severe primary diseases of the heart, brain, liver, kidney, and haematopoietic system, liver function alanine aminotransaminase (ALT) or aspartate aminotransferase (AST) value > 1.5 times the upper limit of the normal value, or abnormal renal function
- Depression or anxiety disorders
- Pregnant or lactating women
- Nervous system conditions, mental illness, or lack of cooperation with the study
- Surgery during the past 4 weeks
- Bleeding tendencies, disseminated intravascular coagulation (DIC), abnormal international standard ratio (INR) value, or thrombocytopenia
- Participation in another trial in the past 1 month
- Allergies to the test drug or an allergic constitution
- Aphasia that affects data collection
- Other obvious TCM syndrome

##### Rejection criteria

The rejection criteria are as follows:

- Reluctance to continue the trial
- Unwillingness to take the trial drug
- No medical record by which to be evaluated

##### Suspension criteria

The study may be suspended for the following reasons:

- Non-cooperation of the participants during the trial for any reason
- The decision to suspend the study by CFDA or other authority
- Occurrence of serious adverse events (SAEs)
- Discovery of a deviation in the protocol

### Randomisation

A total of 120 eligible participants will be allocated to either the XFZYC group or placebo group in a 1:1 ratio using a block randomisation method. The central randomisation system will be used and performed by the Clinical Evaluation Centre of China Academy of Chinese Medical Sciences, which will also maintain the randomisation lists.

### Blinding

All participants, investigators, hospital pharmacist, project inspectors, statisticians and other staff will be blinded to the treatment assignment of the participants. The researchers of the Clinical Evaluation Centre will be responsible for blinded pairing of drugs and participants and will keep emergency envelopes to be opened in the event of medical emergency or severe adverse events during the trial.

### Sample size

Due to the lack of preliminary studies on the evaluation of the efficacy of XFZYC for QS&BSS using the traditional Chinese medicine patient-reported outcome (PRO) scale, we could not calculate the appropriate sample size. Therefore, we adopted this pilot study with an appropriate sample size of 60 participants per group. The outcomes of this trial will provide data support for the calculation of the appropriate sample size for future clinical trials.

### Intervention

**Fig. 1:**
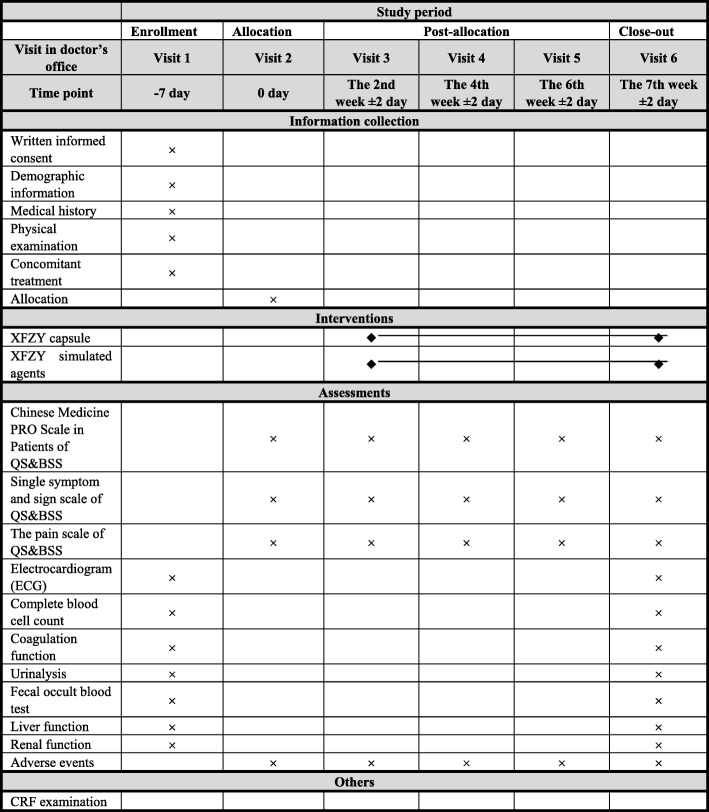
Content for the schedule of enrolment, interventions and assessments. CRF, case report form

The participants in the test group will take six granules of XFZYC (0.4 g/granule) twice a day for 7 weeks. The participants in the control group will take six granules of an XFZYC-simulated agent (0.4 g/granule) twice a day for 7 weeks. All drugs will be taken orally after meals. The XFZYC and XFZYC-simulated agents will be provided by Tianjin Hongrentang Pharmaceutical Co., Ltd., Tianjin, China. Although the colour and smell of the contents of XFZYC and XFZYC-simulated agents are not identical, the capsule shell makes the drugs seem less different. All the drugs are concealed in the same sealed and opaque packages. The label of the package contains the drug name, the approval number, functions, usage, dosage, storage conditions, expiration date, and the name of the manufacturer. Participants will be informed that they would be randomly assigned to receive either XFZYC or XFZYC-simulated agents. They will be encouraged to contact the investigators if they have any uncomfortable feeling or if they think the drugs are not helpful. Participants in both groups can continue their prior routine treatments, with the exception of Chinese herbal medicine. The details of these routine treatments will be recorded in the case report forms (CRFs). Details of the study procedures are shown in Fig. 1.

### Outcome measures

The primary objective of this trial is to assess the feasibility and acceptability of a range of outcome measurements to determine the appropriate ones for future definitive trials. Data completeness ≥ 80% is required for an outcome to be considered for definitive trials. Reported and recorded values will be considered complete, while unknown and bland values will be considered missing:

1. The enrolled participants will be provided with the traditional Chinese medicine PRO scale to assess the change in the clinical symptoms of QS&BSS [21] (http://kns.cnki.net/) (See Additional file 3: Table S2).
2. Each patient’s single symptom and sign evaluation will use the score of the single symptom and sign scale of QS&BSS (See Additional file 3: Table S3). According to the degree of symptoms and signs, the score will be assigned as follows: no = 0; mild = 1; moderate = 2; or severe = 3 [22].
3. Pain levels will be assessed using the score of the pain scale of QS&BSS (See Additional file 3: Table S4). The total score includes a visual analogue scale pain index [23] and the score of degree, duration, and frequency of pain [24]. The location of the pain will be recorded independently.

Each of the above scales will be completed on the day of enrolment and at the 2nd, 4th, 6th, and 7th weeks. Among the multiple time points for the measurements, we will mainly focus on the data from the day of enrolment and the 7th week, which are the intended measurement time points according to previous studies. Additionally, we will observe the “inflexion point”, which refers to the time point when the clinical symptoms of QS&BSS changes during the intervention, to optimise the measurement time point for an adequately powered definitive trial in the future.

### Safety and adverse events

Participants will receive the following tests on the day before enrolment and the day after completion of the intervention period:

1. Physical examination (temperature, respiration, heart rate, blood pressure, height, and weight)
2. Electrocardiogram (ECG)
3. Complete blood cell count
4. Coagulation function (prothrombin time (PT), activated partial thromboplastin time (APTT), thrombin time (TT), fibrinogen (FIB), D-dimer, INR)
5. Urinalysis
6. Faecal occult blood test
7. Liver function (ALT, AST, alkaline phosphatase (ALP), serum total bilirubin (STB), and γ-glutamyl transpeptidase (γ-GT))
8. Renal function (creatinine (Cr), blood urea nitrogen (BUN), and urine N-acetyl-β glucosaminidase)

Any adverse events will be recorded in a CRF with details, including occurrence time, severity, duration, effective measures and the outcomes. According to the judgement of severity, the investigators will determine whether the participants should be suspended or withdrawn from the trial. When SAEs occur, such as severe haemorrhage, hepatic failure, renal failure or death, the investigator will report to the principal unit and ethics committee, and the investigator can open the patient’s emergency envelope to determine the group allocation.

### Data management and statistical analysis

The clinical data management system (http://www.tcmcec.net/) will be used to collect and manage the data. All data from the completed CRFs will be imported into the system twice by two independent data entry clerks to ensure the accuracy. The database will be locked when the principal investigators and statisticians check the data. The original medical records will be archived and saved in the unit responsible for 5 years after the completion of the trial.

All statistical analyses will be performed by an independent statistician blinded to the allocation, using the Statistical Analysis System (SAS, Version 9.2, Institute Inc., Cary, NC, USA) and assuming a two-sided test and a 0.05 level of significance. Based on the strict principle of intention-to-treat (ITT), the outcome measurements will be analysed using a full analysis set (FAS), which means the data of the participants will be analysed according to their original allocation, regardless of the treatment they actually received, withdrawals, losses to follow up, or crossovers. The last-observation-carried-forward (LOCF) method will be used for missing data, and safety outcomes will be analysed using safety sets (SS). Quantitative data will be analysed using the *t* test and analysis of variance (ANOVA). Enumeration data will be analysed using the chi-square test or non-parametric tests. We will use the paired *t* test to analyse significant differences between pre and post treatment. The total dropout rate in each group will be analysed using the chi-square test or Fisher’s exact test. Moreover, the reasons for dropouts will also be descriptively analysed and compared between the two groups. For the safety analysis, the incidence rate of adverse events will be evaluated using the chi-square test and described in a list.

### Quality control

Quality control will be performed as follows

1. Request for the investigators:

The investigators must possess the qualifications and abilities needed to conduct the trial and will not be continually changed. The principal investigators will ensure the trial is conducted according to the standard operating procedure. A project manager at each site will be responsible for the quality of the research.

2. Quality control throughout the entire process of the trial:

Before the trial, investigators will receive pre-trial training on patient screening, data filling, drug use, adverse event (AE) reporting and other matters. During the trial, the investigators will confirm that the participants have received the drugs on time and record the receipt on the medication record form. Those patients who do not take the drugs at the appropriate time will be encouraged to do so, and their reasons will be recorded in detail. Trial supervisors will visit each site regularly to check the case report forms and informed consent to ensure that the trial is strictly following the protocol. The investigators will take measures (to schedule further visits and follow-ups) to keep the incidence rate of dropout within 20%. The number of withdrawals, dropouts, and cases of compliance will be recorded in detail. The trial data will be entered into the database within 2 weeks of being collected. After the trial, all the data will be checked by the quality controller, principal investigator, and statisticians [2].

## Discussion

The research design of a previous study pertaining to XFZYC was characterised by the objectives of patients with QS&BSS with only one simple disease, and they were expected to evaluate whether the addition of XFZYC to the standard routine treatment would enhance the effectiveness and improve the relevant biomedical parameters. However, this trial is focused on the outcome of *Zhengs*, and we chose the PRO scale of QS&BSS to assess the improvement of relevant symptoms and signs. We will also compare the outcomes among different diseases by subgroup analysis. When the evidence accumulates to a certain degree that supports this treatment approach and theory, TCM complementary and alternative treatments will be available to a wider range of the world’s population and will be more pragmatic and standardised.

What might be the mechanism underlying the use of XFZYC for patients with different diseases? To date, several studies have found that QS&BSS was associated with traits such as haemodynamics, haemorheology, neuroendocrine function, vascular endothelial cells, blood lipids, inflammatory substances, genomics, proteomics, and metabolomics, which manifest with a decrease in peripheral vascular compliance and tissue plasminogen activator (t-PA) activity, an increase in plasma neuropeptide Y and tumour necrosis factor-α (TNF-α), interleukin-6 (IL-6), a change in the expression of microR-7641 and micR-4484, plasma protein CD44SP, or a change in the metabolism of glucose, arachidonic acid and linoleic acid [3, 25–30]. Based on the biological essence of the *Zheng* of QS&BSS, other studies have illustrated that XFZYC enhances anti-atherogenic activity, improves cardiac microcirculation, prevents platelet aggregation, modulates angiogenesis, potentiates t-PA, reduces inflammatory markers, and offers neuroprotection [31–36]. We can infer that QS&BSS may be related to the common pathogenesis of several types of diseases that might be treated by XFZYC.

This trial has limitations. First, the trial will be conducted only in Beijing, and whether the results could be generalized to the rest of mainland China is uncertain. Second, this is the first trial in China that focuses on the outcome of *Zhengs*; thus, there is a lack of previous data, which means that the sample size could not be calculated and that this study is a pilot study. Third, although the evaluation of the outcomes of *Zhengs* is a common method of clinical outcome evaluation in TCM, this method has rarely been used in published studies because *Zhengs* have not been clearly defined or understood. Therefore, more clinical studies are required to confirm the eligibility and accuracy of this method.

In conclusion, this trial is the first study designed to demonstrate the outcomes of *Zhengs* of XFZYC in the treatment of QS&BSS patients.

### Trial status

The trial started recruiting on 6 May 2017 and will be completed by December 2018.

## Additional files

Additional file 1: Standard protocol items: recommendation for interventional trials (SPIRIT) 2013 checklist: Recommended items to address in a clinical trial protocol and related documents. (PDF 171 kb)

Additional file 2: **Figure S1.** Study flow chart. (PDF 203 kb)

Additional file 3: **Table S1.** TCM syndrome diagnostic scale of QS&BSS. **Table S2.** Chinese Medicine PRO Scale in Patients of Qi-stagnation and blood-stasis Syndrome. **Table S3.** Single Symptom and Sign Scale of QS&BSS. **Table S4.** Pain Scale of QS&BSS (PDF 360 kb)

## Abbreviations

ALP: Alkaline phosphatase
ALT: Alanine aminotransaminase
APTT: Activated partial thromboplastin time
AST: Aspartate aminotransferase
BUN: Blood urea nitrogen
CFDA: China Food and Drug Administration
CHM: Chinese herbal medicine
CONSORT: Consolidated Standards of Reporting Trials
Cr: Creatinine
CRF: Case report form
DIC: Disseminated intravascular coagulation
FIB: Fibrinogen
GCP: Good Clinical Practice
INR: International standard ratio
PRO: Patient-reported outcome
PT: Prothrombin time
QS&BSS: Qi stagnation and blood stasis syndrome
RCT: Randomised clinical trial
SAEs: Serious adverse events
SPIRIT: Standard protocol items: recommendation for interventional trials
STB: Serum total bilirubin
TCM: Traditional Chinese medicine
TT: Thrombin time
XFZYC: Xue-Fu-Zhu-Yu capsule
γ-GT: γ-Glutamyl Transpeptidase
