# Supplementary figures and images for: Xue-Fu-Zhu-Yu capsule in the treatment of qi stagnation and blood stasis syndrome: a study protocol for a randomised controlled pilot and feasibility trial
Source: Trials. 2018 Sep 21;19:515. doi: 10.1186/s13063-018-2908-9 (PMC6151047; doi:10.1186/s13063-018-2908-9)

Figure S1 Study flow chart

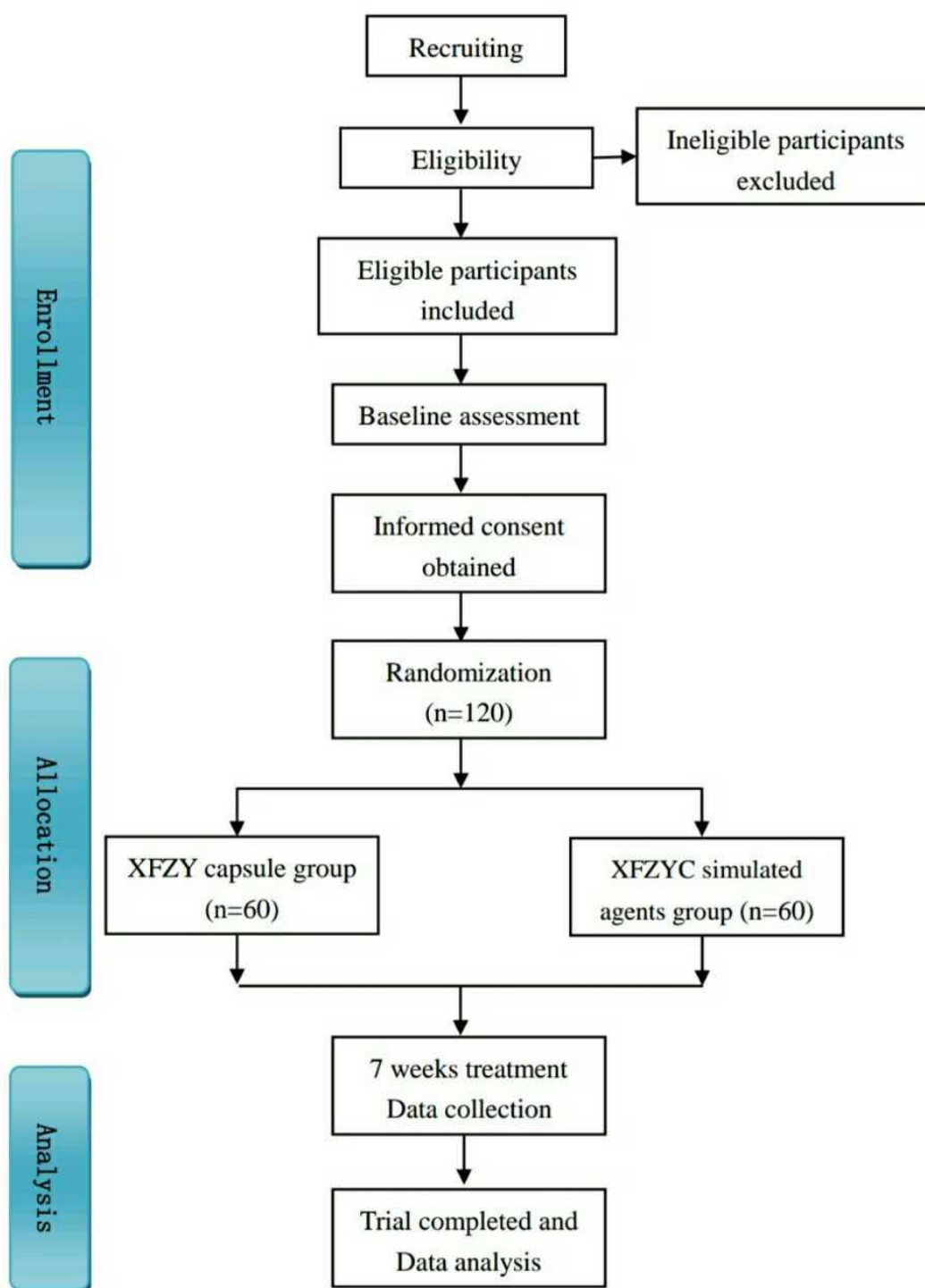

Supplement: Supplementary file 2 — Figure S1. Study flow chart. (PDF 203 kb) [file 13063_2018_2908_MOESM2_ESM.pdf]
