# Supplementary material for: Xue-Fu-Zhu-Yu capsule in the treatment of qi stagnation and blood stasis syndrome: a study protocol for a randomised controlled pilot and feasibility trial
Source: Trials. 2018 Sep 21;19:515. doi: 10.1186/s13063-018-2908-9 (PMC6151047; doi:10.1186/s13063-018-2908-9)
Supplement: Supplementary file 3 — Table S1. TCM syndrome diagnostic scale of QS&BSS. Table S2. Chinese Medicine PRO Scale in Patients of Qi-stagnation and blood-stasis Syndrome. Table S3. Single Symptom and Sign Scale of QS&BSS. Table S4. Pain Scale of QS&BSS (PDF 360 kb) [file 13063_2018_2908_MOESM3_ESM.pdf]

Table S1 TCM syndrome diagnostic scale of QS&BSS

Symptoms/signs	Score		Score
	Yes	No	
**Pain**	9	0	
**Irritability/depression**	16	0	
**Distending pain**	2	0	
**Scurry pain**	6	0	
**Chest distress**	0.5	0	
**Lumps in body**	7	0	
**Petechia in the tongue**	4	0	
**Purplish tongue**	1	0	
**Unsmooth pulse**	4	0	
**Deep pulse**	2	0	
**Total score**			
**if≥20 points, diagnosed as QS&BSS**			

Notes：pian include stomachache, abdominal pain, low back pain, dysmenorrhea, breast pain, limb pain, etc.

Table S2 Chinese Medicine PRO Scale in Patients of Qi-stagnation and blood-stasis Syndrome

Instruction:

In this questionnaire, you are going to answer 37 questions, which include physiology, psychology, independence and society four aspects. Please answer the question according to your specific situation for the last 2 weeks, and check in the□ correspondently. This questionnaire applies five-points scoring method,

for example: Do you feel chest pain? □None □Occasionally □Sometimes(Usually) □Frequently □Always

Thank you for your cooperation!

I. Physiological domain

1. Do you feel distending or stabbing pain on your body?

□None □Occasionally □Sometimes(Usually) □Frequently □Always

2. Is that pain fixed?

□None □Occasionally □Sometimes(Usually) □Frequently □Always

3. If you press on the painful area, will you feel aggravatingly painful?

□None □Occasionally □Sometimes(Usually) □Frequently □Always

4. Is there any lump on your body?

□None □Occasionally □Sometimes(Usually) □Frequently □Always

5. Are stringy veins easy to be notice?

□None □Occasionally □Sometimes(Usually) □Frequently □Always

6. Have you ever notice the cyanotic lips?

□None □Occasionally □Sometimes(Usually) □Frequently □Always

7. Is there any ecchymosis on the skin?

□None □Occasionally □Sometimes(Usually) □Frequently □Always

8. Have you ever feel abdominal distension in the last 2 weeks?

□None □Occasionally □Sometimes(Usually) □Frequently □Always

9. Have you ever feel short of breath in the last 2 weeks?

□None □Occasionally □Sometimes(Usually) □Frequently □Always

10. Have you ever notice any abnormal complexion changes?

□None □Occasionally □Sometimes(Usually) □Frequently □Always

11. Do you have dysmenorrhea? (only for women)

□None □Occasionally □Sometimes(Usually) □Frequently □Always

12. Do you have a irregular menstruation? (only for women)

□None □Occasionally □Sometimes(Usually) □Frequently □Always

13. Do you have any blood loss experience?

□None □Occasionally □Sometimes(Usually) □Frequently □Always

14. Do you eat less than normal?

□None □Occasionally □Sometimes(Usually) □Frequently □Always

15. Have you ever feel belch or hiccough?

□None □Occasionally □Sometimes(Usually) □Frequently □Always

16. Do you feel dizziness?

□None □Occasionally □Sometimes(Usually) □Frequently □Always

17. Have you ever notice any dry, rough and lusterless with red speckles and purple macules on your skin?

□None □Occasionally □Sometimes(Usually) □Frequently □Always

18. Have you ever feel dry in the mouth but unwilling to drink?

□None □Occasionally □Sometimes(Usually) □Frequently □Always

19. Do you feel eyes dull?

□None □Occasionally □Sometimes(Usually) □Frequently □Always

20. Do you feel uncomfortable on the hypochondrium?

□None □Occasionally □Sometimes(Usually) □Frequently □Always

21. Do you have any sensation of oppression on the chest?

□None □Occasionally □Sometimes(Usually) □Frequently □Always

22. Do you have any bitter taste in the mouth?

□None □Occasionally □Sometimes(Usually) □Frequently □Always

II. Psychological domain

23. Do you have any irascibility rashness or impatience?

□None □Occasionally □Sometimes(Usually) □Frequently □Always

24. Do you have any kind of depression?

□None □Occasionally □Sometimes(Usually) □Frequently □Always

25. Do you usually sigh?

□None □Occasionally □Sometimes(Usually) □Frequently □Always

26. Do you always regard life as an interesting journey?

□None □Occasionally □Sometimes(Usually) □Frequently □Always

27. Are you confident to welcome your future life?

□None □Occasionally □Sometimes(Usually) □Frequently □Always

28. Do you have faith in conquering diseases?

□None □Occasionally □Sometimes(Usually) □Frequently □Always

29. Can you keep concentrating on a specific thing?

□None □Occasionally □Sometimes(Usually) □Frequently □Always

30. Are you satisfied with your health condition?

□None □Occasionally □Sometimes(Usually) □Frequently □Always

31. Will your diseases push you into spiritual pain?

□None □Occasionally □Sometimes(Usually) □Frequently □Always

III. Independence

32. Will the signs and symptoms affect your daily life or work?

□None □Occasionally □Sometimes(Usually) □Frequently □Always

IV. Social domain

33. Do you regard yourself as a burden on family members or friends?

□None □Occasionally □Sometimes(Usually) □Frequently □Always

34. Does your disease affect on your family economic status?

□None □Occatinally □Sometimes(Usually) □Frequently □Always

35. Do you feel satisfied with medical environment or traffic environment?

□None □Occatinally □Sometimes(Usually) □Frequently □Always

36. Do you trust your doctor?

□None □Occatinally □Sometimes(Usually) □Frequently □Always

37. Do you think the symptoms affect your social activity?

□None □Occatinally □Sometimes(Usually) □Frequently □Always

Usage of the PRO scale: According to the scale framework, this scale can be scored in 4 domains. The options from "none" to "always" are given 1-5 points of the all above entries except 36th item. The options of 36th entry, from “none” to “always”

are given 5-1 points. Because the number of entries and the highest score in each domain are different, it's not conducive to compare the total score between domains. So, we use standard score (SS) to stand for the scores of each domain. The sum of the actual score (AS) of each entry divided by the total score of each entry in the domain, then multiplied by 100, is the SS of each domain. SS = $(S_1+S_2+\ldots+S_n)$ / $(n*5)*100$. E.g. in Physiological domain, the number of entry is 22, the AS are $S_1+S_2+\ldots+S_{22}$, so the SS are $(S_1+S_2+\ldots+S_n)$ / $(22*5)*100$.

Table S3 Single Symptom and Sign Scale of QS&BSS

Symptom/sign	No(0)	Mild(1)	Moderate(2)	Severe(3)
Pain				
Headache				
Stomachache				
Abdominal pain				
Low back pain				
Joint pain				
Scurry pain				
Irritability				
Depression				
Sigh				
Lumps in body				
Blood spot under the				
Cyanosis of skin				
Limitation of activity				
Chest distress				
Flustered				
Rib-side distension				
Gastrectasia				
Abdominal distension				
Acid flux				
Anorexia				
Insomnia				
Dizziness				
Week				
Fatigue				
Thirst				
Numbness				
Cyanosis of nail				
Dim complexion				
Scaly dry skin				
Purple and dark lip				

Tongue and pulse	No(0)	Yes(1)
Dark red tongue		
Purplish tongue		
Petechia in the tongue		
Thin and whitish fur		
Thin and yellowish fur		
Umsmooth pulse		
String like pulse		
Deep pulse		

Table S4 Pain Scale of QS&BSS

**Position of pain:**	
**Visual Analogue Scale pain index:**	score
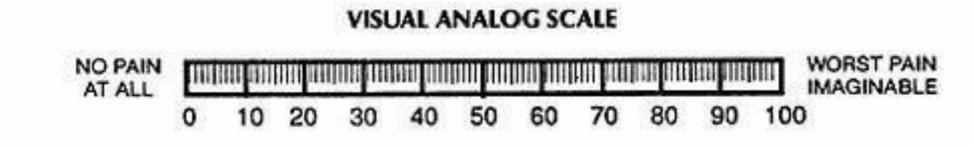 	
**Degree of pain**	0 score：None
	2 score：Mild pain(doesn’t affect work and life, VAS score 1-3)
	4 score：Moderate pain(affects work and life partly, VAS score4-6)
	6 score：Severe pain(affects work and life, need rest, VAS score 7-10)
**Duration of pain**	0 score：None
	2 score：Less than 1 hour / day
	4 score：1-2 hours / day
	6 score：Over 2 hours / day
**Frequency of pain:**	1-7 score: day/week
**Total scores of pain:**	
